# Correlation between Multilocus Sequence Typing and Antibiotic Resistance, Virulence Potential of *Campylobacter jejuni* Isolates from Poultry Meat

**DOI:** 10.3390/foods11121768

**Published:** 2022-06-15

**Authors:** Xiang Wang, Qiyun Zhuo, Yi Hong, Yufan Wu, Qiang Gu, Dawei Yuan, Qingli Dong, Jingdong Shao

**Affiliations:** 1 School of Health Science and Engineering, University of Shanghai for Science and Technology, Shanghai 200093, China; xiang.wang@usst.edu.cn (X.W.); zhuoqy0119@163.com (Q.Z.); 15951073898@163.com (Y.H.); qdong@usst.edu.cn (Q.D.); 2 Centre of Analysis and Test, School of Chemistry and Molecular Engineering, East China University of Science and Technology, Shanghai 200237, China; 3 Technology Center of Zhangjiagang Customs, Suzhou 215600, China; respro@163.com (Q.G.); ydw811210@126.com (D.Y.)

**Keywords:** *Campylobacter jejuni*, MLST, antibiotic resistance, virulence, foodborne pathogen, food safety

## Abstract

*Campylobacter jejuni* is a major foodborne pathogen and can be transmitted to human beings via the consumption of poultry products. This study aimed to determine antibiotic resistance and virulence potential of one hundred *C. jejuni* isolates from poultry meat and to explore the correlation between them and the multilocus sequence types (MLST). A total of 29 STs and 13 CCs were identified by MLST, of which 8 STs were first identified. The dominant ST was ST583 (21%), followed by ST42 (15%), ST61 (12%), and ST2276 (10%). Eighty-eight isolates showed resistance to at least one antibiotic. The resistance rate to fluoroquinolones was the highest (81%), followed by tetracycline (59%), whereas all the isolates were susceptible to erythromycin and telithromycin. Multi-antibiotic resistance was detected in 18 *C. jejuni* isolates. Great variability in the adhesion and invasion ability to Caco-2 cells was observed for the 100 isolates, with adhesion rates varying between 0.02% and 28.48%, and invasion rates varied from 0 to 6.26%. A correlation between STs and antibiotic resistance or virulence was observed. The ST61 isolates were significantly sensitive to CIP, while the TET resistance was significantly associated with ST354 and ST6175 complex. ST11326 showed substantially higher resistance to gentamicin and higher adhesion and invasion abilities to Caco-2 cells. The results helped improve our understanding of the potential hazard of different genotypes *C. jejuni* and provided critical information for the risk assessment of campylobacteriosis infection.

## 1. Introduction

*Campylobacter jejuni* is an important foodborne pathogen causing bacterial gastroenteritis in human beings and a significant health burden worldwide [1]. Contaminated poultry meat is regarded as the primary source of *C. jejuni* infection. Although *C. jejuni* is insignificant for poultry health, it becomes highly invasive upon colonization of the human intestinal tract causing severe [2]. Usually, campylobacteriosis is a self-limiting disease with fever, diarrhea, and headache symptoms. But sometimes, infections still lead to arthritis, septicemia and Guillain-Barre Syndrome (GBS) [3,4].

Antibiotic resistance and virulence are two of the most important biological characteristics of *C. jejuni*. Virulence is one factor that determines the pathogenicity of *C. jejuni* to the host; resistance to antibiotics increases the difficulty of infection treatment. Antibiotic is generally considered for the treatment of *C. jejuni*. However, with the spread of these antibiotics implemented in clinical and animal husbandry, *C. jejuni* isolates have obtained apparent resistance and gradually enhanced [5,6]. Moreover, symptoms caused by *C. jejuni* are also related to its virulence. The pathogenesis of *C. jejuni* infection is not entirely clearly understood, but flagella-mediated motility, adherence to intestinal epithelial cells, invasion, and survival in the host cells, as well as toxins production, are known as essential virulence factors [7,8]. Adherence to and invasion of host cells are two key steps in bacterial pathogenesis, deserved to explore the mechanisms of the two steps. In vitro models of *C. jejuni* infecting human and animal cells have helped to determine the ability of adhesion and invasion [3,9].

Due to the increased sporadic outbreaks, a reliable typing method is necessary to characterize *C. jejuni* strains and investigate the epidemiology of *C. jejuni* infections [10]. Typing has demonstrable value in monitoring *C. jejuni* outbreaks and examining genetic diversity. There are various genotyping methods used for molecular differentiation of *C. jejuni*. Among them, multilocus sequence typing (MLST) has been the most widely applied due to its high discriminatory power and reproducibility. MLST is an extensively used bacterial typing method of *C. jejuni* that displays a reasonable level of *C. jejuni* sequence types (STs) [11]. STs of *C. jejuni,* which are closely related, were classified as one clonal complex (CC), defined as groups of at least two individual isolates with an ST sharing identical alleles at four or more loci out of 7 [12]. It was reported that CC-353 and CC-464 are the most frequently reported *C. jejuni* genotypes in chicken meat in Central China [5], CC-21, accounted for 39.3% of the whole *C. jejuni* isolates, was the major type in East China [13].

Various studies on *C. jejuni* suggested the high genetic and phenotypic diversity, which indicated that not all isolates or genetic lineages pose equal risks to human health [14,15,16]. Isolates of different subgroups may exhibit diverse biological characteristics. For example, *C. jejuni* isolates of ST21 (CC21) possess the most virulence-associated genes among 23 STs studied [5]. In addition, the strong correlation between isolates of ST22 and GBS outbreaks has been widely reported [14,16,17]. Significant associations between specific clonal complexes and human infection may imply the correlation between genotype and pathogenicity [18,19,20]. Moreover, a few studies on *C. jejuni* also demonstrated that antibiotic resistance was associated with ST types. In particular, quinolone resistance was significantly associated with ST353 complex [21,22], ST6411 was associated considerably with ciprofloxacin (CIP)-nalidixic acid (NAL)-tetracycline (TET)- streptomycin (STR) antibiotic resistance pattern [23].

In the present work, the epidemiology of 100 *C. jejuni* isolates from poultry meat in East China was analyzed using MLST. Antibiotic susceptibility and Caco-2 cell assay were conducted to determine antibiotic resistance patterns and virulence potential, respectively. On this basis, the correlation between MLST typing and the two biological characteristics of *C. jejuni* isolates was analyzed.

## 2. Materials and Methods

### 2.1. Bacterial Strains and Genomic DNA Extraction

One hundred strains isolated from poultry meat samples in East China and the reference *C. jejuni* (NCTC11168) strain were used in this study. The *C. jejuni* stock cultures were maintained at −80 °C in brucella broth (Beijing Luqiao Co., Beijing, China) with 10% sheep blood and 15% glycerol. The strains were first activated and inoculated on Columbia blood plate and incubated at 42 °C for 48 h under microaerobic conditions (85% N_2_, 10% CO_2_, 5% O_2_) [24]. The cultures were then activated a second time in Mueller Hinton Broth (MH, Beijing Luqiao). To extract genomic DNA, a bacterial pellet was obtained by centrifugation of 1.5 mL aliquot cultured bacterial suspension at 8000 g for 10 min. The genomic DNA of the isolates was extracted using QIAamp DNA mini kit (QIAGEN) according to the manufacturer’s instructions.

### 2.2. MLST Analysis

Seven housekeeping genes (*aspA*, *glnA*, *gltA*, *glyA*, *pgm*, *tkt*, and *uncA*) were used in the MLST analysis, performed according to the protocol described previously [25]. The nucleotide sequences of the amplified genes were determined by commercial service (Shenggong, Shanghai, China). The alleles and the ST were carried out by submitting the sequences to the pubMLST database (https://pubmlst.org/campylobacter/, accessed on 20 January 2022), which was based on the nucleotide sequencing of 7 housekeeping genes of *C. jejuni*: *aspA* (aspartase), *glnA* (glutamine synthetase), *gltA* (citrate synthase), *glyA* (serine hydroxy methyl transferase), *pgm* (phosphor glucomutase), *tkt* (transketolase), and *uncA* (ATP synthase α subunit) [25]. For novel alleles and STs that were not included in the database, gene sequences were submitted to the pubMLST database for assignment of new allele and MLST type number.

### 2.3. Adherence and Invasion to Caco-2 Cells

Adherence and invasion assays were performed using human colonic carcinoma (Caco-2) cells. Caco-2 cells were maintained in Dulbecco’s modified Eagle’s medium (DMEM) (Life technology, New York, NY, USA) supplemented with 10% fetal bovine serum (FBS), 1% streptomycin-penicillin solution, and 1% nonessential amino acids. The cells were grown in a 50-cm^2^ flask at 37 °C in a humidified 5% CO_2_–95% air incubator until cell layers were confluent [26]. Adherence and invasion assay was performed as previously described with modifications [27]. Caco-2 cells were transferred into 12-well tissue (10^7^ cells per well) culture plates and incubated for 40 h in the conditions described above until cells’ growth reached confluence. *C. jejuni* culture, grown in MHB at 42 °C to stationary phase, was centrifuged and resuspended in 1 mL DMEM to obtain a bacterial suspension of OD_600_ of 0.1, which was roughly equal to 10^8^ CFU/mL of cells [28]. The suspension was inoculated into 12-well tissue culture plates containing confluent monolayers of Caco-2 cells. The specific number of inoculated bacteria was determined by serial dilutions and plate counting. Infected plates were incubated in 5% CO_2_ at 37 °C for 2 h to allow bacteria to adhesion and invasion. For adhesion after 2 h of inoculation, each well was washed twice with 1× phosphate-buffered saline (PBS) in the plate, the cell monolayer was peeled off from the bottom of the 12-well plates with pancreatin (0.25%) and lysed with 1% Triton X-100 for 5 min before enumeration. For invasion, Caco-2 cells were incubated for 2 h at 37 °C and 5% CO_2_ and then t treated with 1 mL of the DMEM containing 100 μg/mL streptomycin-penicillin for 1 h at 37 °C in 5% CO_2_, aim to kill non-invasive bacteria. The cells were washed with 1× PBS twice and treated with pancreatin and Triton X-100 as described above. The bacteria associated with the cells (intracellular and extracellular bacteria) were determined by plate counting. The assay was performed for at least two biologically independent replicates. The results were presented as percentages (mean ± standard deviation) of adhering and internalized *C. jejuni* cells compared to the starting inocula [29].

### 2.4. Antibiotic Susceptibility Testing

Evaluation of antibiotic susceptibility of the isolates was performed using in vitro broth microdilution assay according to the harmonized rules for the monitoring and reporting of AMR in Europe (Commission Implementing Decision 2013/652/EC) [30]. Briefly, the colonies were grown on Columbia agar for 24 h, and Mueller Hinton Broth supplemented with blood (Oxoid, Basingstoke, UK) was used to make a bacterial suspension of 0.5 McFarland (equivalent to 1.5 × 10^8^ CFU/mL). Then, using the Sensititre system (Thermo Fisher Scientific, Dardilly, France) the broths were separately dispensed into Camp2 microtiter plates (Thermo Fisher Scientific) containing known scalar concentrations of the following antibiotics: azithromycin (AZI, 0.015–64 μg/mL), nalidixic acid (NAL, 4–64 μg/mL), ciprofloxacin (CIP, 0.015–64 μg/mL), clindamycin (CLI, 0.03–16 μg/mL), tetracycline (TET, 0.06–64 μg/mL), florfenicol (FEN, 0.03–64 μg/mL), gentamicin (GEN, 0.12–32 μg/mL), erythromycin (ERY, 0.03–64 μg/mL), and telithromycin (TEL, 0.015–8 μg/mL). After inoculation, the plates were incubated at 42 °C in a microaerophilic atmosphere for 24 h and then screened. To evaluate the MICs of the isolates, Swin v3.3 Software (Thermo Fisher Scientific) was used in accordance with the epidemiological cutoff values (ECOFFs) as defined by EUCAST (European Committee on antimicrobial breakpoints) to interpret their antimicrobial susceptibilities. *E. coli* ATCC 25922 was used as a control bacterium in the testing. MIC breakpoints of resistance were >0.5 μg/mL for CIP, >0.25 μg/mL for AZI, >4 μg/mL for ERY, >2 μg/mL for GEN, >16 μg/mL for NAL, >1 μg/mL for TET, >0.5 μg/mL for CLI, >4 μg/mL for TEL and >4 μg/mL for FEN. Multi-drug resistance (MDR) of the isolates was defined as resistance to three or more antibiotic categories.

### 2.5. Statistical Analysis

Statistical analysis of the correlation was determined by SPSS 25.0 (SPSS Inc., IBM Corporation, Armonk, NY, USA). The correlation between MLST complex and antibiotic resistance was performed using the chi-squared test. For the ST type containing five or more isolates (expected value), Pearson’s chi-squared test was used; Fisher’s exact test was used for isolates number less than 5 [22,23]. One-way ANOVA was used to explore whether there was a difference in virulence potential (adherence and invasion capability) among STs. Correlation between MLST complex and pathogenic ability were performed using the Eta-squared. The probability value of *p* < 0.05 was considered to be statistically significant.

## 3. Results

### 3.1. MLST Genotypes of C. jejuni

Sequence types (STs) are considered to belong to the same clonal complex (CC) when they share alleles at four or more of the seven MLST loci. The assignment of STs and CCs was performed by PubMLST (pubmlst.org/neisseria/) web sites. The reference strain *C. jejuni* NCTC 11168 belongs to ST 43. From the assembled contigs of 100 *C. jejuni* isolates, 75 (75%) isolates were assigned to 18 distinct sequence types (STs) grouped into 13 clonal complexs (CCs), while 25 isolates belong to 11 STs without a defined CC (Table 1). The most prevalent STs were ST583 (21 isolates), ST42 (15 isolates), ST61 (12 isolates), and ST2276 (10 isolates), each of the remaining STs containing less than 10 isolates. Although none of a new allele was found, eight of the *C. jejuni* STs with 13 isolates identified were novel and have not yet been reported elsewhere, the allelic profiles of the new identified ST were shown in Table 2. The most prevalent CCs detected were CC45, CC42, CC61 and CC21, accounting for 64% of the isolates. Two of the novel STs (ST11322 and ST11326) were assigned to the CC353 and CC464, respectively, the others (ST11148, ST11149, ST11150, ST11321, ST11325 and ST11327) belong to none of the existing clonal complex reported. 

### 3.2. Antibiotic Susceptibility of C. jejuni Isolates

The *C. jejuni* isolates were analyzed for antibiotics susceptibilities to nine antibiotics using the broth microdilution methods. Overall, the antibiotic resistance profiles showed 13 isolates (including NCTC 11168) were sensitive to all antibiotics tested, 88 isolates exhibited resistance phenotypes to at least one antimicrobial agent, among which 28 isolates showed resistance to one or two tested antibiotics, 18 isolates exhibited multi-antibiotic resistance (MAR) which are resistant to at least three antibiotic categories (Table 3). Most of the isolates were resistant to CIP (81%), NAL (77%) and TET (59%). In contrast to the high levels of fluoroquinolone and tetracycline resistance, *C. jejuni* isolates showed a low level of resistance to CLI (9.9%), GEN (8.9%), AZI (7.9%) and FEN (1.0%). Moreover, all *C. jejuni* isolates were sensitive to ERY and TEL. For MDR isolates, the most prevalent resistant pattern was CIP-TET-NAL, which was observed for 38 isolates. One isolate was resistant to a maximum of 5 antibiotics (CIP-TET-FEN-NAL-CLI). The results demonstrated that *C. jejuni* isolates are highly resistant to antibiotics, particularly to fluoroquinolone and tetracycline.

### 3.3. Associations between MLST Genotypes and Antibiotic Resistances

**Figure 1:**
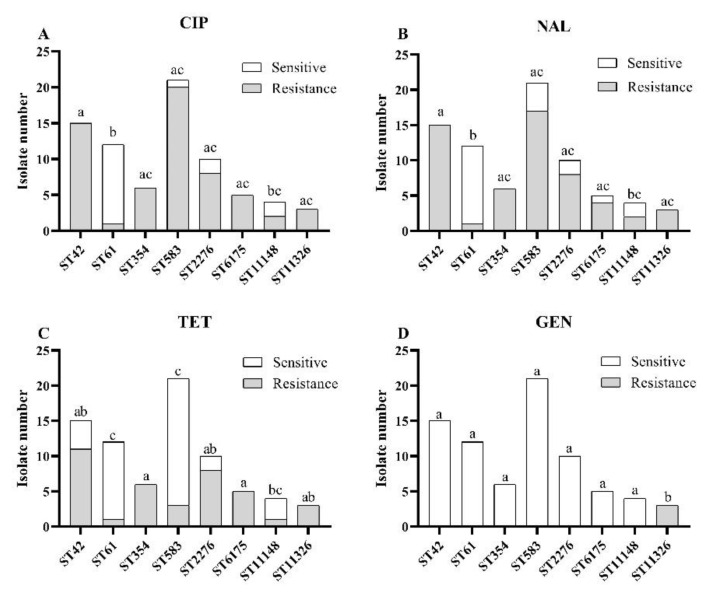
Correlations between STs and resistance to four antibiotics. (**A**) CIP; (**B**) NAL; (**C**) TET; (**D**) GEN. The same letter on column represents no significant difference (*p* ≥ 0.05); a different letter represents a significant difference between STs (*p* < 0.05).

The correlation between STs and antibiotic resistance of *C. jejuni* isolates was analyzed and shown in Figure 1. Eight STs that included at least three isolates were used to perform the correlation analysis. There was a significant correlation between the STs and resistance to four antibiotics (CIP, NAL, TET, GEN) among the nine tested. For resistance to fluoroquinolones antibiotics (CIP and NAL), isolates of ST61 were most sensitive, while isolates of ST42, ST354, ST583, ST2276, ST6175 and ST11326 mainly were resistant. The chi-square test showed that the isolates from the ST61 showed significantly lower (*p* < 0.05) resistance than the other STs except for ST11148 (Figure 1A,B). Furthermore, ST61, ST583 and ST11148 were correlated with a lower resistance rate to TET (Figure 1C), while the other STs showed a relatively higher resistance rate to TET. Notably, among 76 isolates of the eight STs, only the isolates of the ST11326 (novel ST found in this study) showed resistance to GEN (Figure 1D). 

### 3.4. The Adherence and Invasion to Caco-2 Cells by C. jejuni Isolates 

**Figure 2:**
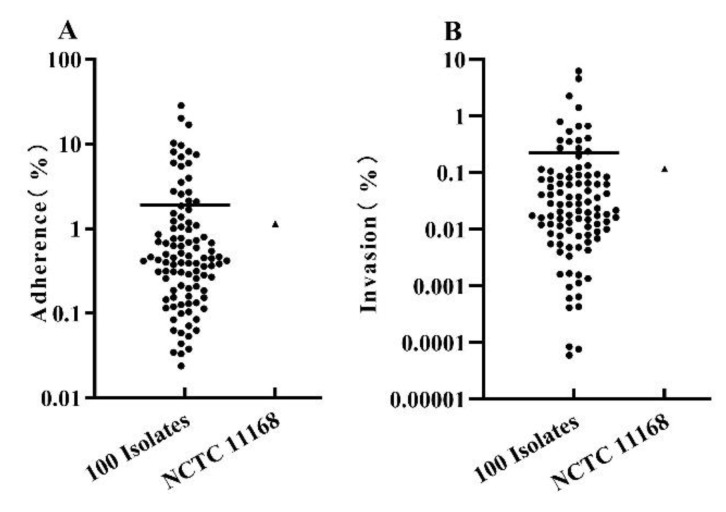
Adherence (**A**) and invasion (**B**) rate of *C. jejuni* to Caco-2 cells.

The pathogenic properties of *C. jejuni* strains isolated from poultry meat were evaluated by adherence and invasion assays to Caco-2 epithelial cells. Compared to the initial infection dose, the results were displayed as percentages of adhering and internalized *C. jejuni* cells. The adhesion rate reflects the ability of bacteria to adhere to the cell surface. Of the 100 *C. jejuni* isolates tested, the adherence abilities varied greatly, ranging from 0.02% to 28.48% with an average of 1.89% (Figure 2A). Most isolates (60/100) adhered to Caco-2 cells within a range of 0.10–1.00%, 23 isolates showed a higher adhesion ability, ranging from 1% to 10%, and 4 isolates showed the strongest capacity for adherence of over 10%, the other 13 isolates showed the lowest level, lower than 0.1%. The process of bacteria migrating cell membrane from cell surface was defined as invasion; the invasion rate means the ability of bacteria to invade the cell. The invasive ability among the isolates ranged from 0.00% to 6.26%, average of invasion was 0.22%. The invasion capability can be divided into four levels as well, most of the isolates (52%) showed invasion capacities between 0.01% and 0.10%, 28 isolates were lower than 0.01%, 16 isolates showed higher invasion capacities ranged 0.1% to 1.0%, and 4 isolates were observed highest invasion percentage (1.42–6.26%). Notably, the isolate that belonged to ST11322 showed the highest capacity of adherence and invasion to Caco-2 cells, with an adherence level of 28.48%, and an invasion level of 4.62%, respectively. As for the reference strain (NCTC 11168), the adherence and invasion rates were 1.14% and 0.12%, respectively, which were both below the average level of 100 isolates (Figure 2).

### 3.5. Correlation between MLST Genotypes and Pathogenic Ability

**Figure 3:**
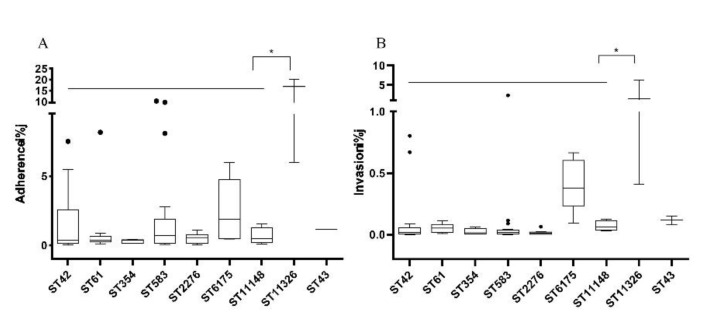
The adherence (**A**) and invasion (**B**) rates of isolates of different STs. The point (•) out of boxes indicates moderated outliers, the line in boxes represents mean value of the STs, the asterisk (*) represents a statistically significant difference (*p* < 0.05) between STs and the adherence or invasion rate.

The correlation between STs and pathogenic ability was shown in Figure 3. ST that included three or more isolates were used for analyses. The *C. jejuni* isolates of different STs exhibited diverse adherence and invasion ability to Caco-2 cells. Overall, significantly higher adhesion and invasion rates of *C. jejuni* were observed for ST11326 isolates (Figure 3). As shown in Figure 2A, isolates belonging to ST11326 showed a mean adherence rate of over 15% for adhesion, while isolates of other STs had a lower Caco-2 cell adhesion rate below 5% on average. Similar results were observed for the invasion ability, a mean invasion rate of 2.7% was observed for ST11326, and less than 0.5% for the other STs.

## 4. Discussion

Poultry and their products are considered as the main cause of human infection with campylobacteriosis [4,6,13]. However, the wide distribution and high molecular diversity among *C. jejuni* make it difficult to compare and identify the source of infection and transmission routes. There are various genotyping methods used for molecular differentiation of *C. jejuni* isolates. Among them, Multilocus sequence typing (MLST) is the most widely applied method for molecular differentiation of *Campylobacter* isolates due to its high discriminatory power and reproducibility [23]. The accumulation of sequence typing data generated from different countries could help to establish a more sophisticated model of epidemiology. Furthermore, the data on antimicrobial resistance and pathogenetic properties are also an indispensable part of it. For these reasons, a wide diversity of *C. jejuni* strains isolated from poultry meat were assessed by MLST in this study, and a correlation analysis was undertaken to determine whether there is a correlation between the resistance or pathogenesis phenotypes and their molecular genotypes.

*C. jejuni* is one of the most common causes of foodborne bacterial diseases reported every year, and reported outbreaks attract much health attention worldwide. The MLST approach can help attribute the infection sources by exploring differences at the molecular level [23]. In the present study, we observed a wide diversity of *C. jejuni* strains circulating in poultry meat, including 18 STs grouped into 13 CCs, and 11 STs without a defined CC. The most predominant type was ST583, which was frequently identified in the *C. jejuni* isolates from poultry-related samples [31,32,33]. The most predominant lineages are CC45, CC42, CC61 and CC21, which accounted for 58% of the 100 isolates. Previous studies have shown that these four clone complexes are widespread in chicken, in samples from other poultry as well as in the isolates from human patients in many countries [14,34,35,36,37]. It was reported that strains of ST45 and ST21 were the most clinically detected [20,38]. Although isolates of these two STs were not found in this study, 30 isolates belonged to the same lineages (CC45 and CC21). ST types belonging to the same CC type are evolutionary and closely related, whether the other STs have the same risk is yet to be explored. Furthermore, clonal complexes such as CC354, CC464, CC574, CC22 and CC353 frequently reported *C. jejuni* genotypes isolated in human disease were also detected in our study. This finding provides evidence of poultry as *C. jejuni* reservoirs and main transmission routes for human infection. In addition, 13 isolates were identified as 8 novel STs: ST11325 (1 isolate), ST11322 (1 isolate), ST11148 (4 isolates), ST11149 (1 isolate), ST11150 (1 isolate), ST11321(1 isolate), ST11326 (3 isolates) and ST11327(1 isolate). These results demonstrated that *C. jejuni* isolated from poultry meat was a heterogeneous population and there may be many new STs remaining to identify.

High antibiotic resistance rates were observed in our study. In all, 88% of the *C. jejuni* isolates were resistant to at least one and 79% against at least two of the tested antibiotics. The isolates kept high levels of resistance to ciprofloxacin (81%), nalidixic acid (77%) and tetracycline (59%). This result is consistent with other studies, that high resistance to nalidixic acid and fluoroquinolones was observed in *C. jejuni* isolates throughout the world [6,39,40]. However, all tested strains were sensitive to ERY and TEL, and showed a low resistance level (8%) to AZI, a similar low-rate was also reported for *C. jejuni* isolated from chicken in EU countries in 2019–2020 [6]. In contrast, a study on 85 *C. jejuni* isolates from chickens and ducks revealed that 68.2% were resistant to ERY [41]. Nowadays, macrolide antibiotics (e.g., AZI, ERY and TEL) has become the preferred drug for treatment of human campylobacteriosis in lots of countries [39,40], the use of this antibiotic should be under a more rigorous attitude. As reported, antibiotic resistance, particularly multi-drug resistance, is apublic health concern [42]. The 18 multi-drug resistant isolates exhibited six resistant profiles, and the most prevalent resistant pattern was CIP-GEN-TET-NAL. This result is not surprising, as fluoroquinolones and tetracycline are commonly used as therapeutic drugs in severe cases of infection in the past decades [43].

Previous studies reported that quinolone resistance was associated with specific ST types [44,45]. Our study explored the correlation between *C. jejuni* STs and antibiotic resistance. A distinct correlation between 8 STs and four antibiotics was observed. Isolates of ST61 were more susceptible to antibiotics CIP, TET, NAL, and GEN than the other STs, while isolates of ST11326 showed a multi-resistant pattern to the above four antibiotics. Habib et al. [32] found that isolates of CC 21 were more resistant to quinolone than those belonging to CC 45. Guyard Nicodème et al. [37] observed that ST-21 isolates showed a higher proportion to ciprofloxacin. However, in the present study, ST6175 and ST 583 which belong to CC 21 and CC 45, respectively, both showed a high level of resistance to quinolone. A more striking finding was the differential association observed in ST11326, which was a novel ST found in this study. A multidrug-resistant pattern CIP-NAL-TET-GEN was observed significantly correlate with isolates in ST11326. A similar find was also reported in Wieczorek’s [23] work, where he showed a significant correlation (*p* < 0.0001) between strains assigned in ST6411 and multidrug-resistant pattern CIP-NAL-TET-STR. 

It is reported that the pathogenic potential of *C. jejuni* is intrinsically linked to the processes of adhesion and invasion in the intestinal epithelium [3]. In the present study, the adherence to and invasion of Caco-2 cells assay were used in our experiments to investigate *C. jejuni* virulence. The consequence displayed a wide range of adherence and invasion rate with an average value of 1.89% and 0.22%, which was higher than the strains from cattle and pig [7]. This may be one of the reasons that poultry is the main source of human infection. *C. jejuni* is an enteric pathogen, which indicates the strong ability to adhere and invade intestinal cells. It was demonstrated that *C. jejuni* showed stronger adhesion and invasion ability to Caco-2 cells than Hela cells [1]. Regarding the virulence potential of a specific isolate, the adherence and invasion ability was inconsistent. For example, the invasion rate of one isolate (ST42) was 0.0004%, and the adhesion rate was as high as 7.50%. A similar phenomenon was demonstrated before [46]. Adhesion can promote the invasion to the host cells but not always lead to invasion. The adherence rate was centered at 0.1% to 1% and the invasion rate was centered at 0.01% to 0.1%. This is in line with previous findings where great variability was observed among isolates [46,47]. 

When taking antibiotic resistance and virulence into consideration, isolates with strong antibiotic resistance and high virulence level pose the highest risk. It is important to note that an isolate belonging to ST11326 possesses a remarkably high rate of adherence and invasion, reaching to 20.11% and 4.62%, respectively. The isolates of ST11326 showed significantly (*p* < 0.05) higher adherence and invasion ability than those of other STs. In addition, they were all multi-antibiotic resistant. The coupling of antibiotic resistance and virulence posed a substantial and alarming issue to food safety and public health. The mechanism behind the coupling is worthy of investigation.

In conclusion, the 100 *C. jejuni* isolates showed high genetic variability in MLST. The overall high resistance rates to antibiotics, especially fluoroquinolones and tetracycline and great variability in adherence and invasion rate were observed. A few STs showed a correlation with antibiotic resistance and virulence potential, which suggested that isolates of different STs may pose distinct risks to human health. These findings suggested that *C. jejuni* genotypes were related to their antibiotic resistance and virulence and could be a clue for *C. jejuni* risk assessment.

## Figures and Tables

**Table 1:** Distribution of clonal complexes (CCs) and sequence types (STs) of 100 *C. jejuni* isolates from poultry meat.

CCs	STs (No. of Isolates)	Total Number
CC45	ST583 (21)	21
CC42	ST42 (15)	15
CC61	ST61 (12)	12
CC21	STST760 (2), ST1811 (1), ST6175 (5), ST6500 (1)	9
CC354	ST354 (6)	6
CC460	ST113 (2)	2
CC464	ST464 (1), ST11325 ^1^(1)	2
CC574	ST2031 (1), ST8149 (1)	2
CC828	ST1586 (2)	2
CC22	ST22 (1)	1
CC353	ST11322 ^1^ (1)	1
CC403	ST8003 (1)	1
CC443	ST51 (1)	1
NA ^2^	ST1962 (1), ST2276 (10), ST4258 (1), ST8881 (1), ST8887 (1), ST11148 ^1^ (4), ST11149 ^1^ (1), ST11150 ^1^ (1), ST11321 ^1^ (1), ST11326 ^1^ (3), ST11327 ^1^ (1)	25

^1^ Novel STs firstly reported. ^2^ STs cannot be divided into any CCs.

**Table 2:** Characteristics of the novel STs identified.

Sequence Type	Clonal Complex	No. of Isolates	MLST Allelic Profile						
			*aspA*	*glnA*	*gltA*	*glyA*	*pgm*	*tkt*	*uncA*
ST11148	NA ^1^	4	1	172	34	4	3	9	6
ST11149	NA	1	9	17	5	10	350	164	3
ST11150	NA	1	37	61	292	545	127	24	35
ST11321	NA	1	8	364	4	470	127	29	35
ST11322	CC353	1	7	114	16	2	2	3	6
ST11325	CC464	1	8	2	2	2	13	3	147
ST11326	NA	3	37	23	263	496	127	1	6
ST11327	NA	1	37	367	292	26	127	57	541

^1^ NA, not assigned to a clonal complex.

**Table 3:** Antibiotic resistance profiles of 100 *C. jejuni* isolates.

Antibiotic Resistance Profiles	NO. of Isolates
Sensitive	12
AZI	2
CIP	4
TET	3
AZI-CLI	1
CIP-TET	1
CIP-NAL	17
CIP-TET-NAL	38
CIP-NAL-CLI	4
AZI-TET-NAL	1
AZI-CIP-TET-NAL	3

## Data Availability

Data are contained within the article.
